# 1-Deoxynojirimycin with theaflavins ameliorates high-fat diet-induced insulin resistance in mice by modulating the gut microbiota

**DOI:** 10.3389/fnut.2025.1690747

**Published:** 2026-02-05

**Authors:** Qiannan Di, Ting Yang, Yiwen Zhao, Wenqing Li, Chunbo Qu, Jingyi Zhang, Yichun Zhu, Chenyang Song, Lixin Na

**Affiliations:** 1 The College of Public Health, Shanghai University of Medicine & Health Sciences, Shanghai, China; 2 Graduate School, Shanghai University of Traditional Chinese Medicine, Shanghai, China; 3 Aerospace Information Research Institute, Chinese Academy of Sciences, Beijing, China

**Keywords:** 1-deoxynojirimycin, gut microbiota, insulin resistance, metabolomics, theaflavins, type 2 diabetes mellitus

## Abstract

**Background:**

This study investigated the ameliorative effects of combined 1-DNJ and TFs on IR in mice through gut microbiota modulation.

**Methods:**

An HFD-induced IR model was established in male mice, which were subsequently divided into Control, Model, 1-DNJ (200 mg/kg·bw/day), TFs (100 mg/kg·bw/day), and 1-DNJ + TFs (200 + 100 mg/kg·bw/day) groups for daily oral administration over 11 consecutive weeks. The ameliorative effects were evaluated by examining biochemical parameters in serum and histopathological changes in the liver pancreatic and colon. Mechanistic insights were elucidated through 16S rRNA gene sequencing of fecal samples and untargeted metabolomics analysis of intestinal contents.

**Results:**

Interventions with 1-DNJ, TFs, or thecombination effectively reduced blood glucose levels and improved insulin sensitivity. The combined treatment demonstrated superior efficacy, decreasing circulating levels of LPS, IL-6, and TNF-α, alleviating hepatic lipid accumulation, and reducing colon tissue barrier damage. Furthermore, the combined intervention profoundly modulated the gut microbiota, characterized by an increased abundance of beneficial bacteria (e.g., Muribaculaceae, Lachnospiraceae_NK4A136_group, Alloprevotella) and a reduction in harmful genera (e.g., Roseburia, Intestinimonas). These microbial shifts were concomitantly associated with significant alterations in intestinal metabolic pathways, including sphingolipid metabolism, necroptosis, glycerophospholipid metabolism, the pentose phosphate pathway, pentose and glucuronic acid interconversion, and tyrosine metabolism.

**Conclusion:**

The combined administration of 1-DNJ and TFs demonstrated superior efficacy in ameliorating HFD-induced IR compared to individual components, with gut microbiota modulation playing a pivotal role. These findings position 1-DNJ and TFs as promising natural candidates for functional food development targeting metabolic health.

## 1 Introduction

Type 2 diabetes mellitus (T2DM), projected to increase 46% from 536.6 million adults in 2021 to 783 million by 2045, is a major global health threat characterized by severe macrovascular and microvascular complications, heightened infection risk, and cognitive impairment (1). Compared to conventional pharmaceuticals, natural active substances derived from foods or traditional medicinal plants are generally perceived to possess superior safety and tolerance profiles. With the emergence of the “food-as-drug” concept (2), research on ameliorating T2DM using natural active compounds has thus become a prominent research focus.

Epidemiological studies have revealed that compared to healthy individuals, significant alterations are observed in the gut microbiota of patients with T2DM (3). Modern research has found that bioactive components derived from traditional Chinese herbs can induce changes in gut microorganisms, characterized by an increase in beneficial bacteria and a decrease in harmful bacteria. Moreover, the beneficial effects of certain key bioactive components on T2DM have been associated with the modulation of the gut microbiota (4, 5). Consequently, targeting the gut microbiota with bioactive components from traditional Chinese herbs to ameliorate diabetes is considered a promising direction for future research.

The complex pathogenesis of T2DM necessitates multi-targeted intervention strategies for effective management. Synergistic combinations of natural bioactive components derived from traditional food-medicine resources often demonstrate superior efficacy. Within the historical context of traditional Chinese medicine, T2DM is categorized as “xiao-ke” (wasting-thirst syndrome) (6). The Compendium of Materia Medica documents both mulberry leaf and black tea as possessing properties to arrest “xiao-ke,” while the Qingnang Secret Transmission specifically describes mulberry leaf-black tea combinations as improving “xiao-ke” symptoms. Modern research has suggested two key components: 1-deoxynojirimycin (1-DNJ), a unique alkaloid in mulberry leaves (7, 8), and theaflavins (TFs), the principal flavonoids in black tea (9, 10). Both exert influences on glucose and lipid metabolism through actions within the intestinal environment, with gut microbiota playing a central role in the mechanisms.

Prior investigations have indicated that 1-DNJ intake is associated with favorable shifts in gut microbial composition, characterized by an increase in beneficial bacteria, which correlates with improvements in glucose and lipid metabolic parameters (11, 12). Similarly, TFs, despite their recognized low systemic bioavailability, are thought to exert substantial blood glucose modulation primarily within the intestinal lumen (13). Crucially, TFs have also been implicated in modulating the gut microbiome to alleviate hyperglycemia and dyslipidemia (14). Given that gut dysbiosis is a recognized factor in T2DM pathogenesis and that multi-targeted interventions often yield superior outcomes, the combined application of mulberry leaf-derived 1-DNJ and black tea-derived TFs presents an nutritional intervention strategy that warrants further investigation. While 1-DNJ and TFs have been shown to independently offer efficacy against insulin resistance through distinct molecular targets, the potential for their combined intervention to produce substantially enhanced anti-diabetic effects via complementary multi-pathway mechanisms and cascade amplification remains largely unexamined. Thus, investigating the gut microbiota-mediated synergy between 1-DNJ and TFs is of significant scientific and clinical importance.

Chronic consumption of a High-Fat Diet (HFD) is widely recognized as a primary environmental driver for insulin resistance (IR) (15–17), which plays a central pathophysiological role in T2DM. In this study, an HFD-induced IR mice model was established to observe the ameliorative effects of 1-DNJ and TFs combination intervention and its influence on gut microbiota. By combining with fecal small molecule metabolite detection, the effects of 1-DNJ combined with TFs on gut microbiota in IR were preliminarily explored from microbial and metabolic perspectives, as well as potential involved biological metabolic pathways. This study provides a theoretical basis for the development of functional foods.

## 2 Materials and method

### 2.1 Animals experiments

The SPF-grade C57BL/6J male mice, aged 4 weeks (weight 14 ± 2 g), were obtained from Changzhou Kavin Experimental Animal Co., Ltd., with an animal license number: SCXK 2021–0013, and an experimental animal use license: SYXK 2018–0029. Animal welfare and experimental procedures were conducted in accordance with the ethical regulations of Shanghai University of Medicine & Health Sciences (NO. 2022SY011). The animals were housed in the Laboratory Animal Center at Shanghai University of Medicine & Health Sciences under standard environmental conditions, which included a 12-h light/dark cycle, humidity maintained at 50 ± 15%, and temperatures controlled at 22 ± 2 °C. Prior to the experiments, the animals underwent a one-week acclimatization period and were fed a regular diet, provided by Slaykang Experimental Animal Co., Ltd., China along with water ad libitum.

All experimental mice were screened for baseline weight and fasting blood glucose before the start of the experiment. Only individuals that met the preset healthy physiological range standards were selected for subsequent studies. Forty male mice were randomly divided into five groups, with eight mice in each group. The groups were, respectively, the control group, the model group, the 1-DNJ, the TFs group, and the 1-DNJ combined with TFs group (1-DNJ + TFs). The mice in the Control group were fed with a regular diet (14.4% fat, 60.0% carbohydrate, 26.6% protein), while the other groups were fed with a high-fat diet (60% fat, 20.0% carbohydrate, 20.0% protein) (18). Concurrently, the 1-DNJ, TFs and 1-DNJ + TFs group were, respectively, administered with 1-DNJ (200 mg/kg), TFs (100 mg/kg), and 1-DNJ + TFs (200 mg/kg + 100 mg/kg) via drinking water for 11 consecutive weeks. The body weight, water intake, and food intake were measured daily. Following a 12-h fast in week 11, successful establishment of the insulin resistance model was defined by fasting blood glucose levels in model mice ranging from 11.10 to 16.00 mmol/L for two consecutive measurements. After the experiment was completed, anesthesia was achieved by intraperitoneal injection of 1.5% pentobarbital sodium. Blood samples were collected by extracting the eyeballs. The collected blood samples were left to stand at room temperature for 30 min, centrifuged at 3500 rpm for 15 min, the upper serum was collected and stored in a − 80 °C refrigerator for subsequent detection. The liver, pancreas, and epididymal adipose tissues were collected. Part of the tissues were placed in 4% paraformaldehyde and fixed at 4 for over 24 h for pathological detection and immunohistochemistry. The remaining tissues were pre-cooled under liquid nitrogen and subsequently stored in a − 80 °C refrigerator for subsequent analysis.

### 2.2 Intraperitoneal glucose tolerance test (IPGTT)

IPGTT were conducted via the intraperitoneal route at predetermined intervals post-subcutaneous transplantation to evaluate the islet response to glucose stimuli. Following an overnight fast, the mice were administered 2 g/kg of a 20% glucose solution intraperitoneally. Blood glucose concentrations were assessed at baseline (prior to injection, designated as time 0 and at 15, 30-, 60-, 90-, and 120-min post-glucose administration). IPGTT levels were measured using a Yuwell glucometer (Yuwell Medical Equipment Co., Ltd., Shanghai, China) following the manufacturer’s instructions.

### 2.3 Serum biochemical indices

Serum levels of total cholesterol (TC), triglycerides (TG), aspartate aminotransferase (AST), and alanine aminotransferase (ALT) were quantified using colorimetric assays. Insulin and glucagon-like peptide-1 (GLP-1), low-density lipoprotein cholesterol (LDL-C), high-density lipoprotein cholesterol (HDL-C) levels, tumor necrosis factor-alpha (TNF-α) and interleukin-6 (IL-6) concentrations were measured via enzyme-linked immunosorbent assay (ELISA). All assays were performed following the manufacturer’s protocols provided in commercial kits (Sangon Biotech Co., Ltd., Shanghai, China). Fasting blood glucose (FBG) levels were measured, and insulin resistance was evaluated using the Homeostatic Model Assessment of Insulin Resistance (HOMA-IR), calculated as follows: HOMA-IR = FBG (mmol/L) × Fasting Insulin (μU/mL)/22.5.

### 2.4 Histopathology histopathological examination

To assess tissue histology, hematoxylin and eosin (H&E) staining was conducted. Tissue sections were first deparaffinized and rehydrated using a graded alcohol series. Subsequently, the sections were stained with hematoxylin for 5 min, differentiated in acid alcohol, and blued in Scotts tap water. Following a distilled water rinse, eosin staining was performed for 3 min. Finally, the sections were dehydrated through a graded alcohol series, cleared in xylene, and cover slipped with a mounting medium.

### 2.5 RNA extraction and quantitative reverse transcription polymerase chain reaction (RT-qPCR)

Tissue samples were homogenized in 1 mL of TRIzol reagent (Vazyme, Nanjing, China) and allowed to stand on ice for 30 min. The chloroform (Sinopharm Chemical Reagent, Ningbo, China) was added and the mixture was vortex and then incubated on ice for an additional 15 min. The homogenate was centrifuged at 12,000 rpm for 20 min to separate the phases. The aqueous phase was carefully aspirated and an equal volume of isopropanol (Sinopharm Chemical Reagent, shanghai, China) was added. The mixture was centrifuged at 8,000 rpm for 10 min to precipitate the nucleic acids. The pellet was retained, washed twice with 75% ethanol, and the RNA was eluted using RNA-free water. The RNA concentration was assessed using a NanoDrop 1,000 spectrophotometer (NanoDrop Technologies, USA).

Total RNA extracted from the samples was reverse transcribed into complementary DNA (cDNA) using the HiScript IV All-in-One Ultra RT SuperMix (Catalog No. R433-01, Vazyme, Nanjing, China), according to the manufacturer’s protocol. qPCR was performed to assess the mRNA expression levels of GLP-1, employing the Taq Pro Universal SYBR qPCR Master Mix, following the guidelines provided in the product manual. Beta-actin served as an endogenous control gene to normalize the expression data. The primer sequences for the genes of interest in this study were as follows: For GLP-1, the forward primer sequence was 5’-TGGACTCCCGCCGTGCCCAA-3′. The beta-actin gene, used as an internal control, had the forward primer sequence 5’-GGAGATTACTGCCCTGGCTC-3′ and the reverse primer sequence 5’-GACTCATCGTACTCCTGCTT-3′.

### 2.6 Western blot

Colonic tissues were lysed with RIPA buffer, and proteins were extracted through high-speed homogenization followed by centrifugation. Protein concentrations were determined using the BCA protein assay. Subsequently, proteins were denatured and equalized in SDS-PAGE loading buffer. SDS-PAGE was employed for electrophoretic separation, and the proteins were transferred to a PVDF membrane by wet blotting. After membrane blocking, incubations with primary and secondary antibodies were performed. The immunoreactive bands were developed using a chemiluminescent substrate and imaged, with *β*-actin as a loading control. The primary antibody used were TNF-α Polyclonal Antibody (Cat. No. 29652-1-AP), IL-6 Polyclonal antibody (Cat. No. 21865-1-AP) and β-actin Polyclonal antibody (Cat No. 20536-1-AP) purchased from Proteintech Group (Wuhan, China). The primary antibody GLP-1(#ab26278) was purchased from Abcam (Cambridge, MA).

### 2.7 16S rRNA sequencing

Microbial DNA was extracted from colonic contents using the DNeasy PowerSoil kit (Qiagen, Hilden, Germany), with DNA integrity assessed by agarose gel electrophoresis for visual quality verification and concentration measured precisely using a NanoDrop spectrophotometer. The V3-V4 hypervariable regions of the bacterial 16S rRNA gene were then amplified by PCR in 25 μL reactions containing universal primer pairs modified with sample-specific barcodes and Illumina sequencing adapters to enable downstream sequencing. Sequences were clustered into Operational Taxonomic Units (OTUs) at 97% similarity, with the most abundant sequences within each OTU selected as representative sequences. Subsequent analyses included taxonomic classification at the species level, quantification of microbial composition, alpha diversity assessments, and community structure characterization.

### 2.8 Metabolomics analysis

Metabolite extraction, sample preparation, and LC–MS/MS analysis were performed following previously published protocols, with raw MS data processed using Compound Discoverer 3.1.0 for preliminary analysis. Ion intensity values were normalized across samples via probability quotient normalization, and metabolite abundance comparisons among the three groups were assessed using one-way ANOVA. Differentially abundant metabolites were identified based on Variable Importance in Projection (VIP) scores derived from partial least squares discriminant analysis (PLS-DA) and orthogonal projections to latent structures discriminant analysis (OPLS-DA) using SIMCA-P 14.0, with metabolites exhibiting a fold change (FC) ≥ 2 or ≤ 0.5, *p*-value < 0.05, and VIP score > 1 subsequently subjected to KEGG pathway enrichment analysis to elucidate biologically relevant metabolic pathways.

### 2.9 Statistical analysis

For statistical analyses, SPSS 23.0 (IBM Corp.) software packages were employed. Data were presented as the mean ± standard deviation (SD). To assess statistical differences across the four groups, one-way analysis of variance (ANOVA) was performed, followed by Least Significant Difference test (LSD) with a 95% confidence interval. In this study, statistical significance was defined as a *p* ≤ 0.05.

## 3 Results

### 3.1 1-DNJ and TFs combination improves IR in mice

**Figure 1:**
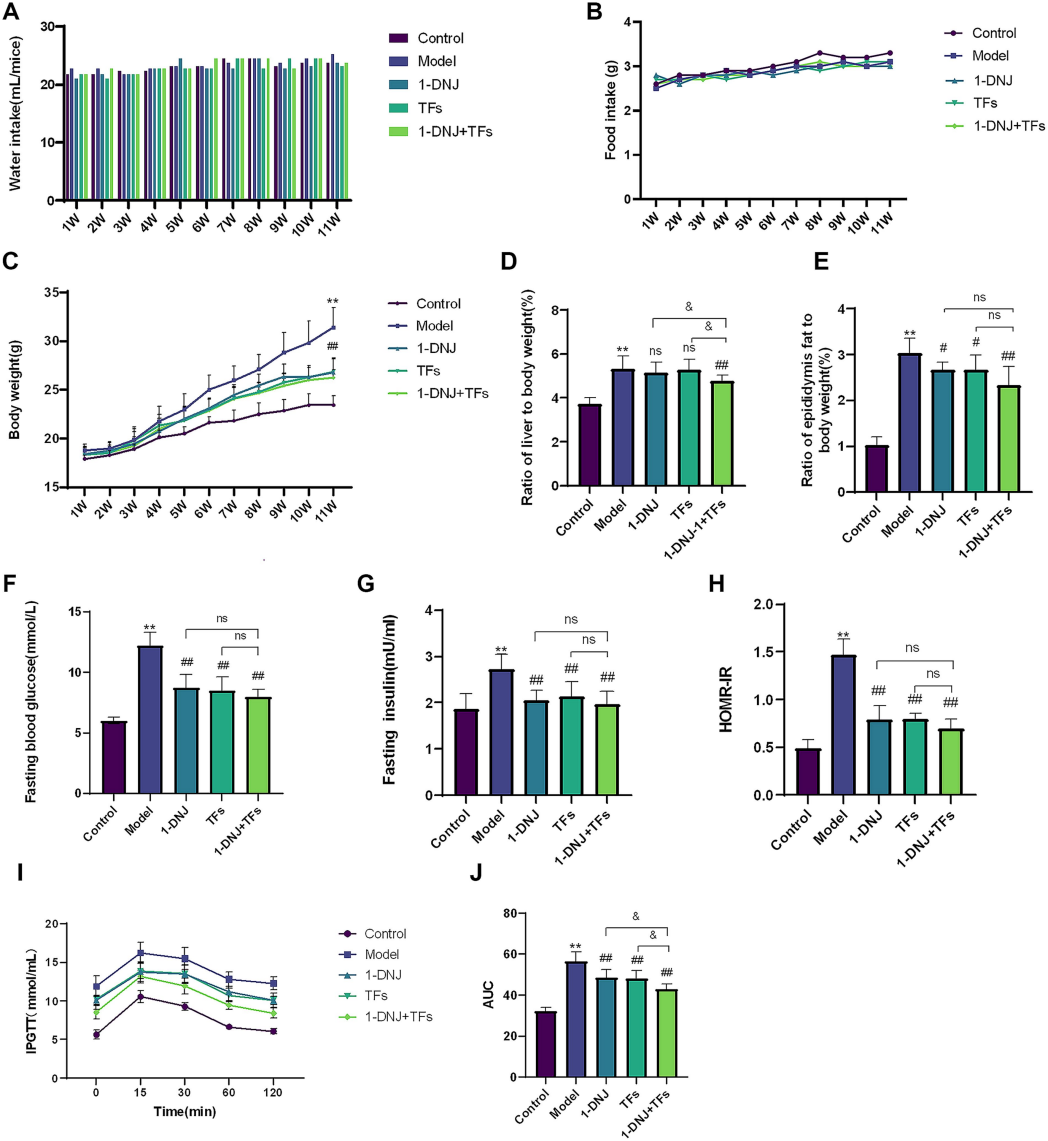
**(A)** Changes in water intake over 11 weeks **(B)** changes in food intake over 11 weeks **(C)** body weight gain of male mice. **(D)** RATIO liver to body weight **(E)** ratio epididymis fat to body weight **(F)** the levels of fasting blood glucose **(G)** the levels of fasting insulin **(H)** the levels of HOMA-IR **(I)** the curve of IPGTT **(J)** an area under the curve of IPGTT. Compared with the control group, **p < 0.01; compared with the model group, #*p* < 0.05 and ##*p* < 0.01; compared with the m1-DNJ + TFs group, &*p* < 0.05, ns: no statistically significant differences between two groups.

Figures 1A,B illustrates comparable 24-h food intake and water consumption across experimental groups, with no statistically significant intergroup differences observed. All intervention groups (1-DNJ, TFs, and 1-DNJ + TFs) effectively suppressed HFD-induced weight gain from week 4 onward. Among these groups, the 1-DNJ + TFs group exhibited the optimal weight-suppressive effect starting from the 6th week (*p* < 0.01), though no significant differences emerged between monointervention and combination groups, indicating equivalent efficacy in counteracting HFD-associated adiposity (Figure 1C). Analysis of organ indices (Figures 1D,E) revealed that the Model group exhibited significantly elevated liver and epididymal adipose tissue indices relative to the Control group (*p* < 0.01).

Regarding hepatic improvement, only the 1-DNJ + TFs group showed a significant reduction in liver index compared to the Model group (*p* < 0.05). In contrast, both single-component interventions (1-DNJ and TFs) significantly reduced epididymal fat mass (*p* < 0.05), with the 1-DNJ + TFs group exhibiting an even more pronounced effect (*p* < 0.01). It was demonstrated that a synergistic combination was pivotal for achieving significant hepatic improvement. Concurrently, both individual components effectively attenuated epididymal fat mass, an effect which was further potentiated by their co-administration. Assessment of glycemic parameters (Figures 1F–H) confirmed that the Model group exhibited significantly elevated FBG, FINS, and HOMA-IR levels compared to the Control group (*p* < 0.01), consistent with an insulin-resistant phenotype. All intervention groups significantly reduced fasting blood glucose levels (*p* < 0.01) while ameliorating compensatory hyperinsulinemia, with the combination group showing superior trends in alleviating insulin resistance. Figures 1I,J revealed that the Model group exhibited a significantly elevated AUC relative to the Control group (*p* < 0.01). All treatment groups significantly lowered AUC values (*p* < 0.01 versus Model), with the combination intervention showing the most pronounced reduction.

**Figure 2:**
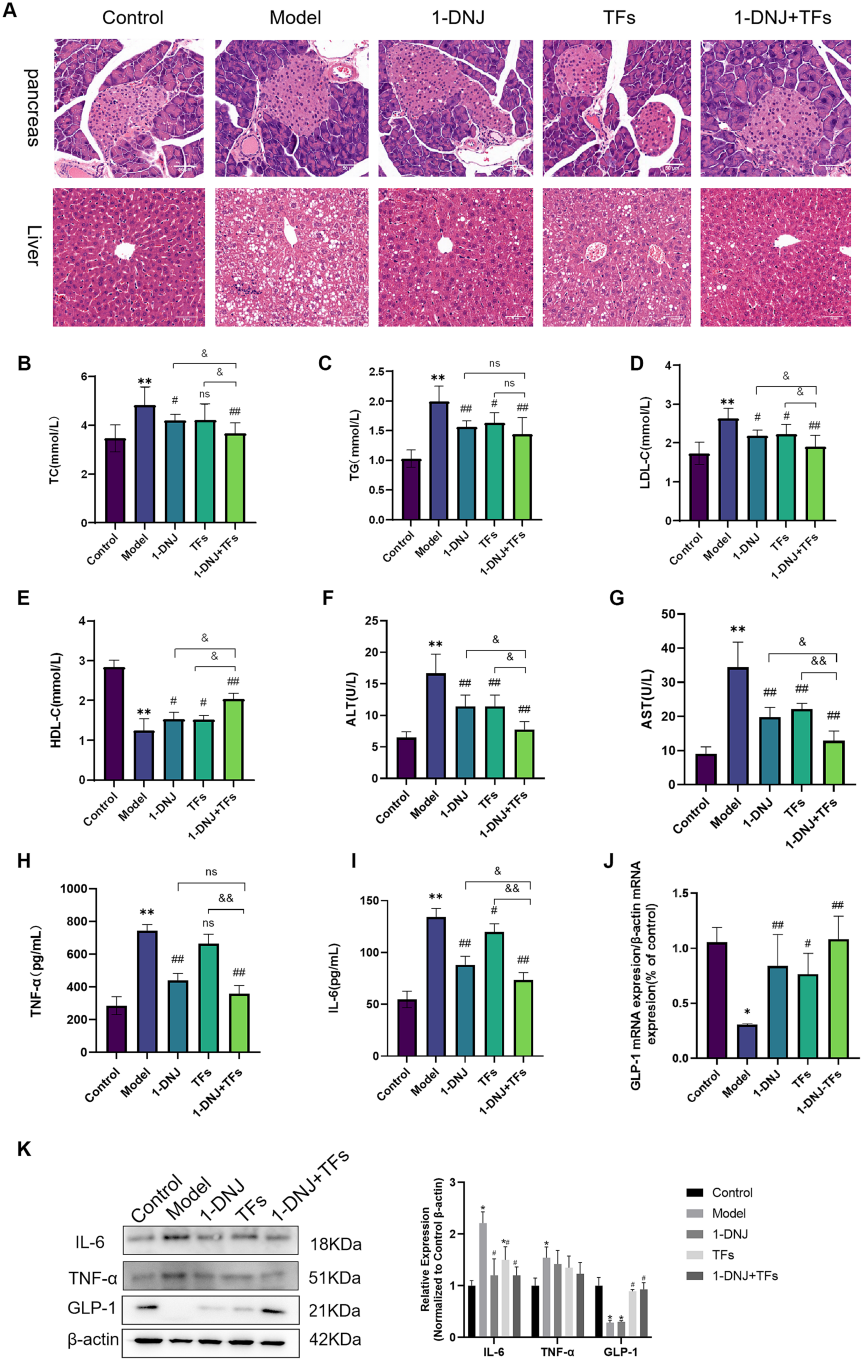
**(A)** Pathological morphologic changes of pancreas and liver via the H&E staining **(B)** the levels of TC in plasma **(C)** the levels of TG in plasma **(D)** the levels of LDL-C in plasma **(E)** the levels of HDL-C in plasma **(F)** the levels of ALT in plasma **(G)** the levels of AST in plasma **(H)** the levels of TNF-α in plasma **(I)** the levels of IL-6 in plasma **(J)** the relative expression of GLP-1 mRNA in colon. **(K)** The expression and grayscale quantification of protein in the colon. Compared with the control group, ^**^p < 0.01; compared with the model group, #*p* < 0.05 and ##*p* < 0.01; compared with the m1-DNJ + TFs group, &*p* < 0.05, ns: no statistically significant differences between two groups.

Histopathological examination of pancreatic and hepatic tissues is shown in Figure 2A. No reduction in islet area or abnormalities in islet morphology were observed in pancreatic tissues across all groups. In the Control group, hepatocytes exhibited a regular radial arrangement centered around the central vein, with normal morphology and orderly alignment, and showed no significant lipid accumulation or inflammatory cell infiltration. The Model group displayed typical pathological features of metabolic liver injury, including disorganized hepatocyte arrangement with disruption of the normal radial structure, along with substantial lipid droplet deposition in the cytoplasm forming characteristic lipid vacuoles. In the 1-DNJ group, hepatocytes showed relatively well-organized alignment with reduced lipid vacuoles. While the TFs group demonstrated improved hepatocyte arrangement, the reduction in lipid vacuoles was less pronounced compared to the 1-DNJ group. Notably, the 1-DNJ + TFs group exhibited the most optimal therapeutic effects, not only maintaining normal hepatocyte alignment but also showing significantly fewer lipid vacuoles compared to either monointervention group. Lipid metabolism analysis (Figures 2B–E) revealed that the Model group had significantly increased TC, TG, and LDL-C, along with reduced HDL-C (*p* < 0.01 vs. Control). Single-component interventions significantly improved TC, TG, LDL-C, and HDL-C levels (*p* < 0.05 vs. Model), whereas the combination group exhibited superior regulatory effects on all lipid parameters (*p* < 0.01), indicating a synergistic hypolipidemic action. As shown in Figures 2F,G, serum AST and ALT levels were significantly elevated in the Model group compared to the Control group (*p* < 0.01). Following intervention with either 1-DNJ or TFs alone, significant reductions in serum AST and ALT levels were observed (*p* < 0.01). Notably, the combination intervention group (1-DNJ + TFs) demonstrated more pronounced improvement in liver function parameters (*p* < 0.01). Regarding inflammatory cytokine levels (Figures 2H,I), serum TNF-α and IL-6 concentrations were markedly increased in the Model group relative to Controls (*p* < 0.01). Both 1-DNJ and TFs monotherapies significantly reduced TNF-α and IL-6 levels (*p* < 0.01). Importantly, the combined intervention exhibited synergistic effects, with superior efficacy in lowering inflammatory cytokines compared to either treatment alone (*p* < 0.01). As demonstrated in Figures 2J,K, serum GLP-1 levels were significantly reduced in the Model group compared to the Control group (*p* < 0.01). Following therapeutic intervention, significant elevations in serum GLP-1 concentrations were observed in both the 1-DNJ and TFs treatment groups relative to the Model group (*p* < 0.01), with the most pronounced enhancement being detected in the 1-DNJ + TFs group (*p* < 0.01). In colonic tissues, both gene and protein expression levels of GLP-1 were significantly downregulated in the Model group compared to the Control group (*p* < 0.05). Following intervention with either 1-DNJ or TFs alone, colonic GLP-1 expression at both transcriptional and translational levels was partially restored (*p* < 0.05). As shown in Figure 2K, compared with the Control group, the protein expression levels of IL-6 and TNF-α in colonic tissues were statistically different in the Model group. The 1-DNJ, TFs, and 1-DNJ + TFs groups exhibited a recovery trend. Specifically, compared with the Model group, the IL-6 protein expression levels in the 1-DNJ, TFs, and 1-DNJ + TFs groups showed significant differences, while no statistical difference was observed in the TNF-α expression level. The results suggested that 1-DNJ and TFs could improve the inflammatory state in colonic tissue, and the combined intervention of 1-DNJ and TFs is even more effective.

### 3.2 1-DNJ and TFs combination improves intestinal barrier integrity in HFD-induced IR mice

**Figure 3:**
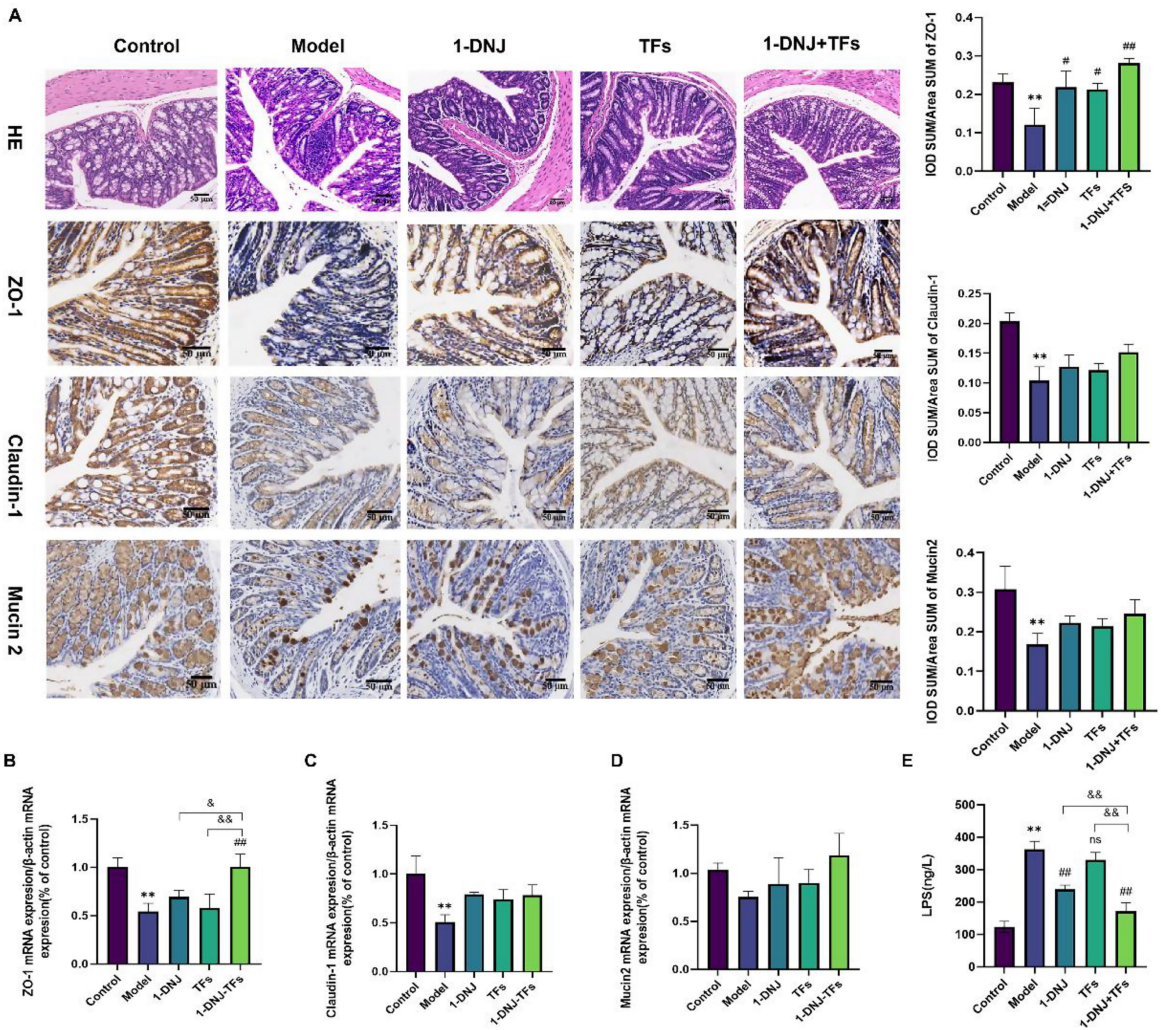
**(A)** Pathological morphological changes of the colon assessed by hematoxylin and eosin (H&E) staining and immunohistochemical staining for ZO-1, Claudin-1, and Mucin 2 in colon sections. **(B)** The relative expression of ZO-1 mRNA in colon. **(C)** The relative expression of Claudin-1 mRNA in colon. **(D)** The relative expression of Mucin2 mRNA in colon. **(E)** The levels of LPS in plasma. Compared with the control group, ^**^p < 0.01; compared with the model group, #*p* < 0.05 and ##*p* < 0.01; compared with the m1-DNJ + TFs group, &*p* < 0.05, ns: no statistically significant differences between two groups.

Histological evaluation (Figure 3A) confirmed preserved mucosal architecture across all experimental groups, with no evidence of epithelial disruption. Notably, inflammatory cell infiltration localized to crypt regions was exclusively observed in the Model group, while treatment groups (1-DNJ, TFs, and 1-DNJ + TFs) maintained intact mucosal morphology. This pattern indicates effective attenuation of HFD-induced colonic inflammation in all intervention group. Immunohistochemical quantification revealed significant downregulation of ZO-1, Claudin-1, and Mucin 2 protein expression in Model group colonic tissues relative to Control (*p* < 0.01). Monointervention with 1-DNJ or TFs significantly restored ZO-1 protein levels versus Model (*p* < 0.05), while combined treatment elicited a more marked increase (*p* < 0.01). At the mRNA level (Figures 3B–D), Model animals exhibited suppressed expression of ZO-1, Claudin-1, and Mucin 2 transcripts compared to Control (*p* < 0.01). While neither monointervention significantly modulated these targets, the 1-DNJ + TFs combination selectively enhanced ZO-1 mRNA expression (*p* < 0.01 vs. Model), in contrast to the limited transcriptional effects of single-agent interventions. Although there were no statistically significant differences in the mRNA and protein expression levels of Claudin-1 and Mucin-2 in the treatment group compared with the model group, an upward trend was observed. Serum LPS quantification (Figure 3E) demonstrated elevated endotoxemia in Model mice versus Control (*p* < 0.01). 1-DNJ monointervention significantly reduced circulating LPS (*p* < 0.01 vs. Model), whereas TFs alone showed no measurable impact. Strikingly, the combination regimen produced the most substantial attenuation of LPS levels, achieving statistical superiority over both Model (*p* < 0.01) and individual treatment groups. In the HFD-induced IR model, the impairment of intestinal barrier function is one of the key links in its pathophysiology. This leads to the continuous translocation of LPS from the intestine into the bloodstream, triggering systemic chronic low-grade inflammation. The results of this study indicate that 1-DNJ and TFs, especially their combined application, repair the intestinal mechanical barrier and reduce intestinal permeability through a multi-pronged approach. Specifically, our intervention upregulated the expression of key tight junction proteins such as ZO-1, which serves as a crucial scaffold protein for tight junctions, and Claudin-1, a vital component that directly contributes to the sealing properties of these paracellular junctions. By enhancing these tight junction proteins, the cellular barrier between epithelial cells is fortified, thereby decreasing the translocation of harmful substances like LPS. Furthermore, our findings also showed restoration trends in the expression of Mucin 2, the major secreted mucin that forms the protective mucus layer—a critical first line of defense that physically separates the intestinal lumen from the epithelial surface and traps microbes. Although the regulatory effects on Claudin-1 and Mucin 2 did not reach statistical significance in all analyses, the consistent restoration of their expression trends also provides potential benefits for improving comprehensive intestinal barrier function.

### 3.3 1-DNJ and TFs combination modulated gut microbiota in HFD-induced IR mice

**Figure 4:**
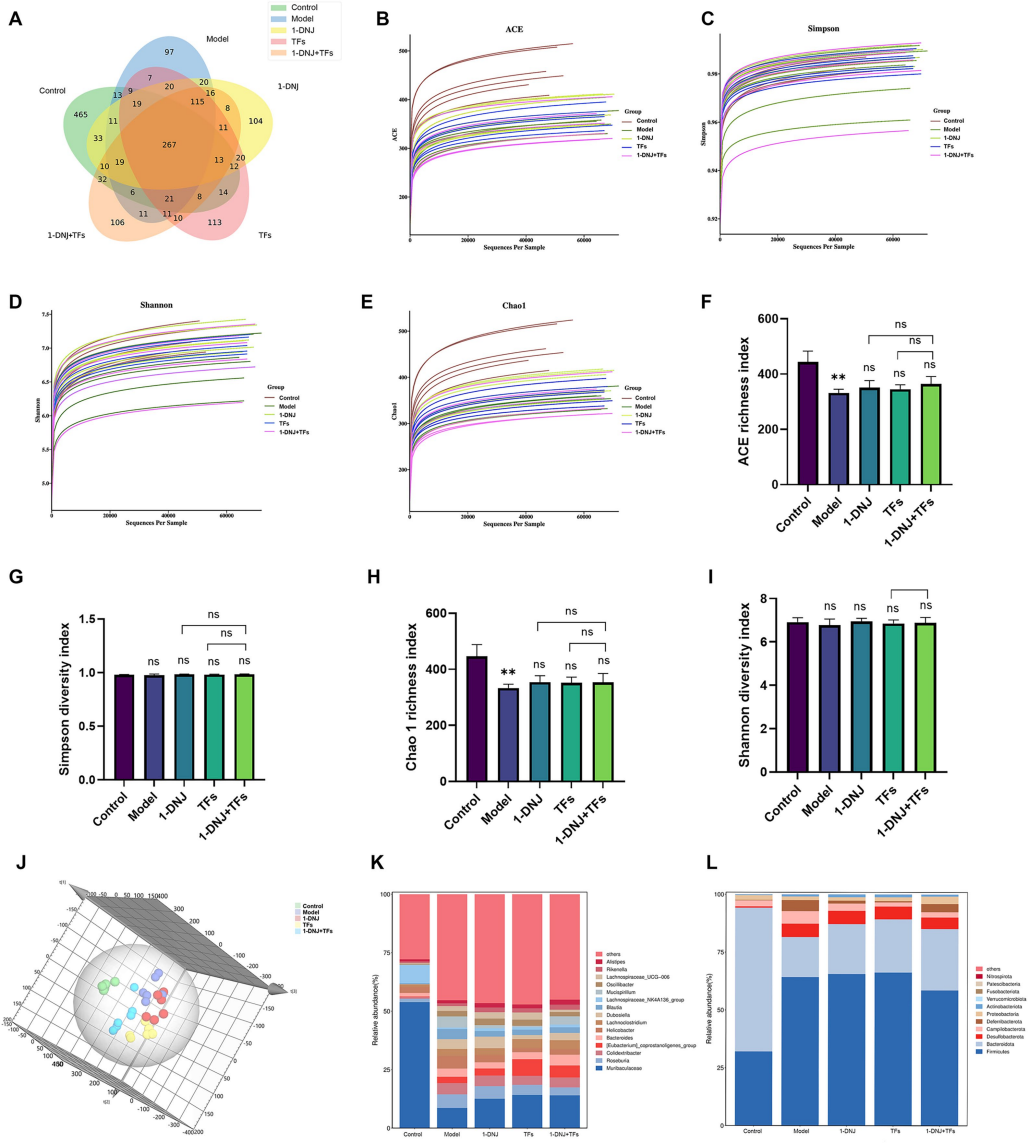
**(A)** Venn diagram of gut microbiota among different groups. **(B)** Rarefaction curves of the ACE index. **(C)** Rarefaction curves of the Simpson index. **(D)** Rarefaction curves of the Shannon index. **(E)** Rarefaction curves of the Chao1 index. **(F)** ACE richness index. **(G)** Simpson diversity index. **(H)** Chao1 richness index. **(I)** Shannon diversity index. **(J)** Inter-group spatial distance matrix of gut microbiota. **(K)** Composition of gut microbiota at the genus level in each group. **(L)** Composition of gut microbiota at the phylum level in each group. Compared with the control group, ^**^p < 0.01; ns: no statistically significant differences between two groups.

The bioinformatics analysis of 16S rRNA gene sequencing data revealed that high-quality sequences were clustered into 1,621 Amplicon Sequence Variants (ASVs) at a 97% similarity threshold following processing by clustering algorithms. There were significant differences in ASV counts among the experimental groups. The Control group exhibited 465 ASVs, whereas the Model group demonstrated a marked reduction to 97 ASVs. Conversely, the intervention groups—1-DNJ, TFs, and the 1-DNJ + TFs groups—displayed 104, 113, and 106 ASVs, respectively, suggesting a trend towards recovery (Figure 4A). As shown in Figures 4B–E, the biodiversity plateau of all samples was observed at approximately 10,000 sequences, and the curves became nearly saturated when sequencing depth reached approximately 40,000 sequences, indicating sufficient sequencing coverage across all samples to capture potential bacterial species for subsequent analyses. In the *α*-diversity analysis of gut microbiota, the ACE richness index (Figure 4F) exhibited the highest value in the Control group, which was significantly reduced in the Model group compared to the Control group. However, no significant differences were observed among the 1-DNJ, TFs, and 1-DNJ + TFs groups relative to the Model group, although an increasing trend was noted. The Simpson diversity index (Figure 4G) showed no significant differences among the Control, Model, 1-DNJ, TFs, and 1-DNJ + TFs groups. The Chao1 richness index (Figure 4H) demonstrated a similar pattern to the ACE index, with the Control group showing the highest value and the Model group exhibiting a significant reduction, while no significant differences were detected between the Model group and the 1-DNJ, TFs, or 1-DNJ + TFs groups. The Shannon diversity index (Figure 4I) also revealed no significant intergroup differences. Collectively, these results indicated that although microbial diversity did not differ significantly among groups, high-fat diet administration substantially reduced microbial richness, with 1-DNJ and TFs interventions showing a tendency to enhance microbial richness. As presented in Figure 4J, principal coordinate analysis (PCoA) revealed distinct separation between fecal microbiomes of all experimental groups, demonstrating notable structural differences in gut microbial communities. The Model group exhibited the greatest distance from the Control group, with the intervention groups positioned between these two extremes, and the 1-DNJ + TFs group showing closer proximity to the Control group. *β*-diversity analysis indicated that combined administration of 1-DNJ and TFs demonstrated superior efficacy in ameliorating high-fat diet-induced T2DM compared to monotherapies with 1-DNJ or TFs alone.

To identify differentially abundant microbial taxa among the combined intervention, single interventions, and Model groups, community structure analysis was performed across all experimental groups. As shown in Figure 4K, at the phylum level, *Bacteroidota* and *Firmicutes* dominated the gut microbiota. Compared to the Control group, *Firmicutes* and *Desulfobacterota* were increased in the Model group, while Bacteroidota was reduced. The 1-DNJ, TFs, and 1-DNJ + TFs groups exhibited elevated proportions of *Bacteroidota*, with the 1-DNJ + TFs group additionally showing reduced Firmicutes levels. At the genus level (Figure 4L), the Model group displayed decreased abundances of Lachnospiraceae_NK4A136_group and *Muribaculaceae*, alongside increased abundances of *Mucispirillum, Roseburia, Colidextribacter, Helicobacter, Lachnoclostridium, Blautia, Dubosiella*, and *Oscillibacter*. Administration of 1-DNJ, TFs, or their combination restored *Muribaculaceae* and *Lachnospiraceae_NK4A136_group* abundances while reducing *Mucispirillum, Helicobacter, Dubosiella, and Oscillibacter* levels. These findings demonstrated that combined 1-DNJ and TFs intervention effectively modulated the gut microbiota structure in T2DM model mice.

**Figure 5:**
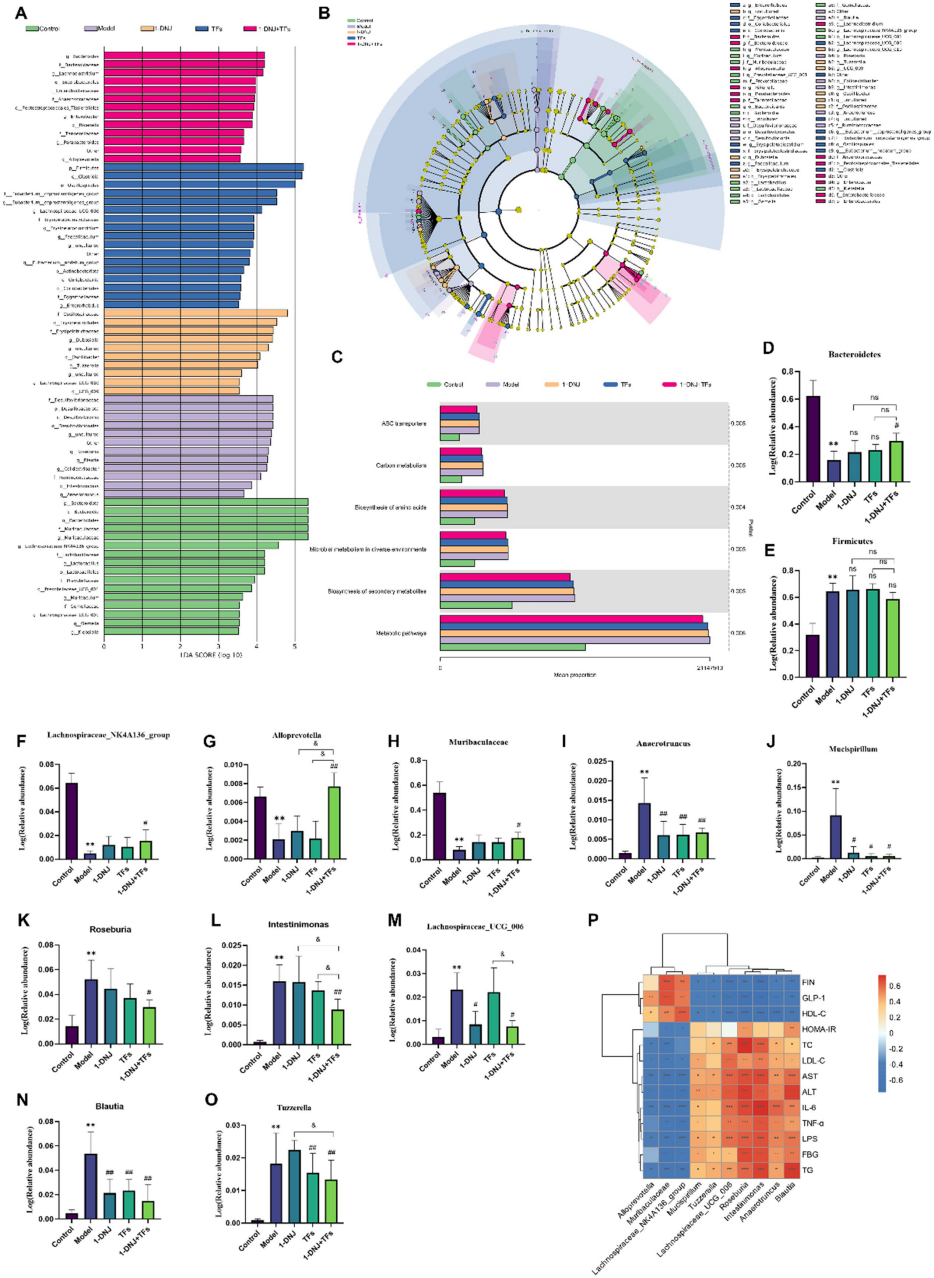
**(A)** LDA score plot of LEfSe analysis. **(B)** Differential species annotation cladogram. **(C)** Predictive functional analysis of gut microbiota. **(D)** Abundance levels of *Bacteroidetes* in each group. **(E)** Abundance levels of *Firmicutes* in each group. **(F)** Abundance levels of *Lachnospiraceae_NK4A136_group* in each group. **(G)** Abundance levels of *Alloprevotella* in each group. **(H)** Abundance levels of *Muribaculaceae* in each group. **(I)** Abundance levels of *Anaerotruncus* in each group. **(J)** Abundance levels of *Mucispirillum* in each group. **(K)** Abundance levels of *Roseburia* in each group. **(L)** Abundance levels of *Intestinimonas* in each group. **(M)** Abundance levels of *Lachnospiraceae_UCG_006* in each group. **(N)** Abundance levels of *Blautia* in each group. **(O)** Abundance levels of *Tuzzerella* in each group. **(P)** Correlation heatmap between differentially abundant bacteria and plasma biochemical indicators in IR. Compared with the control group, ^**^p < 0.01; compared with the model group, #*p* < 0.05 and ##*p* < 0.01; compared with the m1-DNJ + TFs group, &*p* < 0.05, ns: no statistically significant differences between two groups.

To identify characteristic gut microbiota with significant intergroup differences, linear discriminant analysis effect size (LEfSe) was applied to determine taxonomic features that exhibited significant differences among experimental groups. Combined LEfSe analysis was performed to compare dominant bacterial taxa across groups, and taxa with LDA > 3 were presented in Figures 5A,B. Fecal microbiota of the Control, Model, 1-DNJ, TFs, and 1-DNJ + TFs groups exhibited distinct taxonomic profiles, with 7, 5, 5, 6, and 6 identifiable differentially abundant genera, respectively. The Control group was characterized by enrichment of *Lachnospiraceae_NK4A136_group*, *Lactobacillus, Prevotellaceae_UCG_001, Muribaculaceae, Lachnospiraceae_UCG_001, Gemella, and Klebsiella*. The microbial profile of the Model group was significantly associated with *Blautia, Roseburia, Colidextribacter, Intestinimonas, and Anaerotruncus*, suggesting these taxa may contribute to T2DM progression. Administration of 1-DNJ was linked to alterations in *Dubosiella, Oscillibacter, Tuzzerella, Lachnospiraceae_UCG_010, and UCG_009*, while TFs intervention correlated with *Erysipelatoclostridium, Lachnospiraceae_UCG_006, Eubacterium_coprostanoligenes_group, Enterorhabdus, Faecalibaculum, and Eubacterium_nodatum_group*. Combined 1-DNJ + TFs treatment induced significant changes in *Bacteroides, Lachnoclostridium, Alloprevotella, Rikenella, Parabacteroides, and Enterobacter*. These findings demonstrated that combined 1-DNJ and TFs intervention effectively modulated gut microbiota composition in IR model mice.

Further investigation of metabolic functional changes in fecal microbiota induced by combined 1-DNJ and TFs intervention was performed using PICRUSt2 analysis. In the Model group, genes associated with ABC transporters, Carbon metabolism, Biosynthesis of amino acids, Microbial metabolism in diverse environments, Biosynthesis of secondary metabolites, and Metabolic pathways were enriched, indicating significant disruption of these metabolic pathways in IR. Additionally, microbial communities involved in these pathways were altered following combined 1-DNJ and TFs treatment (Figure 5C). These findings collectively demonstrated that gut bacteria participated in systemic metabolic pathway alterations in T2DM, while combined 1-DNJ and TFs intervention restored aberrant metabolic pathways by modulating the abundance of gut bacteria involved in host metabolism.

To further elucidate the microbial basis underlying these metabolic pathway shifts, a genus-level analysis was performed to explore intergroup species differences, focusing on genera that exhibited significant abundance changes in the Model group compared to the Control group and were further modulated by 1-DNJ, TFs, and 1-DNJ + TFs interventions. Ten genera were identified through statistical screening, including *Muribaculaceae, Lachnospiraceae_NK4A136_group, Alloprevotella, Anaerotruncus, Blautia, Intestinimonas, Mucispirillum, Roseburia, Tuzzerella, and Lachnospiraceae_UCG_006*. As shown in Figures 5D–O, compared to the Control group, the Model group exhibited reduced relative abundances of *Muribaculaceae, Lachnospiraceae_NK4A136_group, and Alloprevotella*, whereas the relative abundances of *Mucispirillum, Roseburia, Blautia, Anaerotruncus, Tuzzerella, Intestinimonas, and Lachnospiraceae_UCG_006* were significantly increased. Compared to the Model group, the 1-DNJ, TFs, and 1-DNJ + TFs groups all demonstrated decreased relative abundances of *Anaerotruncus, Mucispirillum, and Blautia*. Furthermore, 1-DNJ specifically reduced Lachnospiraceae_UCG_006 abundance, while TFs reduced *Tuzzerella* abundance. Notably, the 1-DNJ + TFs group exhibited elevated levels of *Lachnospiraceae_NK4A136_group and Muribaculaceae*, with synergistic effects observed for *Alloprevotella* upregulation and *Intestinimonas* downregulation by combined 1-DNJ and TFs intervention. These findings collectively demonstrate that combined 1-DNJ and TFs intervention effectively reversed T2DM-associated reductions in *Muribaculaceae, Lachnospiraceae_NK4A136_group, and Alloprevotell*a relative abundances, as well as the elevated abundances of *Mucispirillum, Roseburia, Blautia, Anaerotruncus, Tuzzerella, Intestinimonas, and Lachnospiraceae_UCG_006*.

Spearman correlation analysis was performed to assess associations between the identified differential genera and serum biochemical parameters. As shown in Figure 5P, *Muribaculaceae, Lachnospiraceae_NK4A136_group*, *and Alloprevotella* exhibited positive correlations with GLP-1 and HDL-C levels, with *Muribaculaceae and Lachnospiraceae_NK4A136_group* also positively correlated with insulin levels. *Muribaculaceae and Lachnospiraceae_NK4A136_group* showed negative correlations with FBG, TC, TG, LDL-C, AST, ALT, TNF-α, IL-6, and LPS levels, while Alloprevotella was not significantly correlated with FBG. *Anaerotruncus, Blautia, Intestinimonas, Mucispirillum, Rosebury, Tuzzerella, and Lachnospiraceae_UCG_006* demonstrated negative correlations with GLP-1, insulin, and HDL-C, but positive correlations with FBG, TC, TG, LDL-C, AST, ALT, TNF-α, IL-6, and LPS. Additionally, *Roseburia and Blautia* were positively correlated with HOMA-IR. These findings indicated that the ameliorative effects of combined 1-DNJ and TFs on IR were associated with modulations in the abundances of *Muribaculaceae, Lachnospiraceae_NK4A136_group, Alloprevotella, Anaerotruncus, and Blautia*.

### 3.4 1-DNJ and TFs combination altered fecal metabolome in HFD-induced IR mice

**Figure 6:**
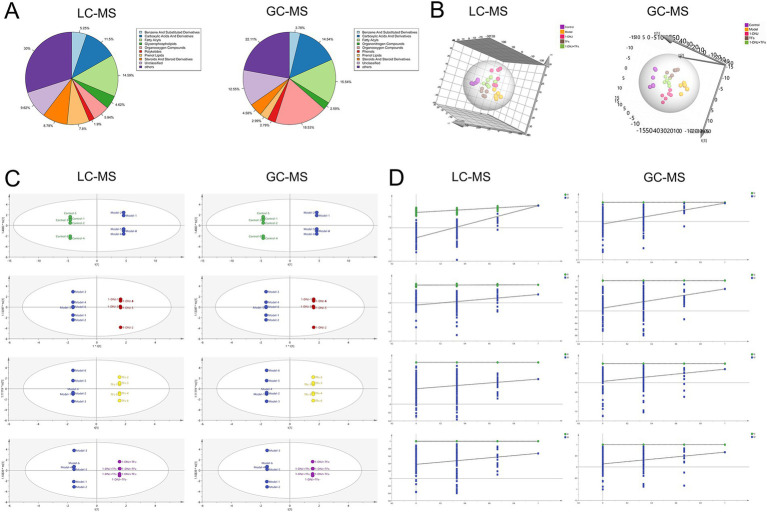
**(A)** Pie charts of metabolite clustering for each group. **(B)** PLS-DA score plot. **(C)** OPLS-DA score plots for each group. **(D)** Permutation test plot for the OPLS-DA model (200 permutations).

To further characterize the metabolic profiles of the intestinal contents, both LC–MS and GC–MS platforms were employed for comprehensive metabolomic analysis. As shown in Figure 6A, the LC–MS analysis revealed that Fatty Acyls were the most abundant metabolite class in the intestinal contents, accounting for 14.59% of all identified metabolites. Benzene and substituted derivatives constituted 11.5%, followed by Steroids and steroid derivatives (8.79%), Prenol lipids (7.8%), Organooxygen compounds (5.94%), Carboxylic acids and derivatives (5.25%), Glycerophospholipids (4.62%), and Polyketides (1.9%). The GC–MS analysis showed a distinct distribution pattern of metabolite classes. Organooxygen compounds were the most prevalent, representing 18.53% of the detected metabolites. Fatty acyls (15.54%) and Carboxylic acids and derivatives (14.54%) were also major components. Additionally, Steroids and steroid derivatives accounted for 4.58%, Prenol lipids for 2.99%, Phenols for 2.79%, and Organonitrogen compounds for 2.59%.

To further elucidate the metabolic alterations induced by different treatments and to assess the discriminatory capacity of the metabolomic profiles, partial least squares discriminant analysis (PLS-DA) was performed on the metabolites detected by the dual-platform LC–MS and GC–MS analyses. The GC–MS-derived model showed strong alignment with the observed data (R^2^X = 0.734, R^2^Y = 0.966) and robust predictive power (*Q*^2^ = 0.695), capturing over 73% of metabolic profile variability while maintaining clear separation between experimental groups. For the LC–MS dataset, the model similarly demonstrated high explanatory capacity (R^2^X = 0.762, R^2^Y = 0.917) and acceptable predictive performance (*Q*^2^ = 0.531), explaining nearly 76% of metabolic variance and fulfilling analytical requirements for group discrimination. In the PLS-DA score plot (Figure 6B), clear separation among all experimental groups was observed, confirming distinct metabolic signatures across the control, model, and treatment groups. Notably, the 1-DNJ, TFs, and 1-DNJ + TFs groups clustered in an intermediate position between the control and model groups, with the 1-DNJ + TFs group showing the closest proximity to the control group. This spatial distribution pattern indicates that both 1-DNJ and TFs could partially restore the metabolic dysregulation induced by the model intervention, and their combined administration exerted the most pronounced therapeutic effect.

**Figure 7:**
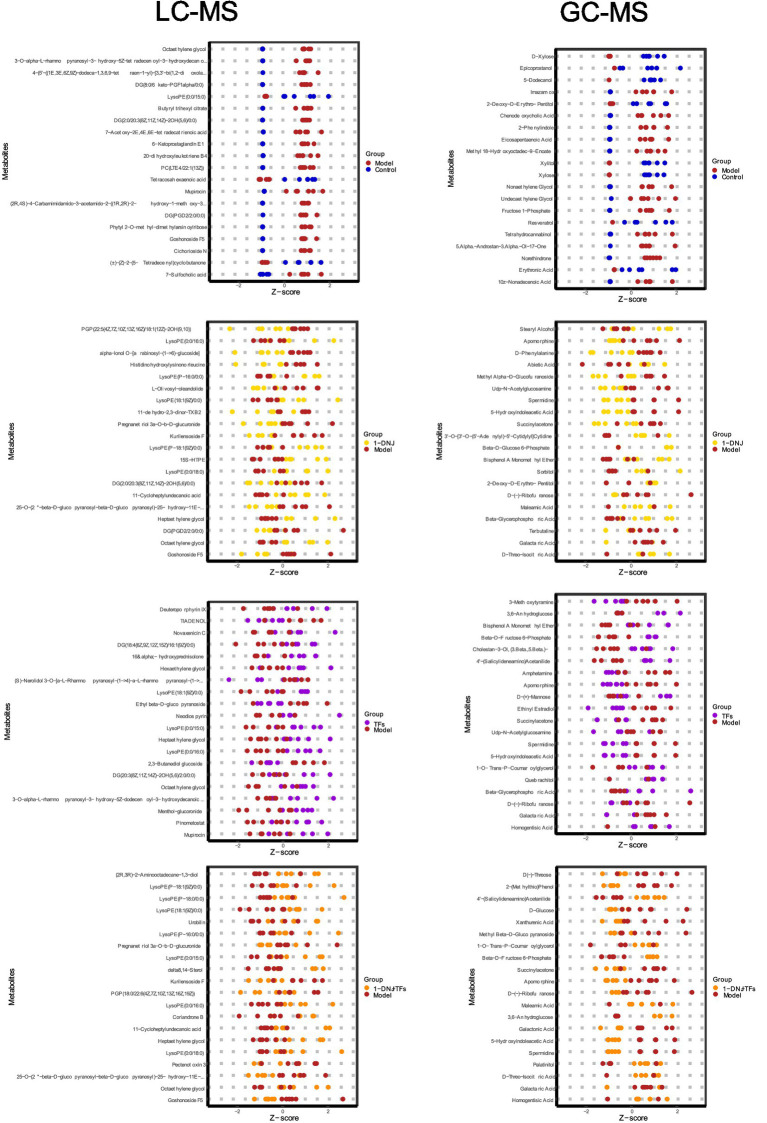
Top 20 differentially abundant metabolites based on VIP values.

To further dissect intergroup metabolic disparities, orthogonal partial least squares discriminant analysis (OPLS-DA) was conducted. As illustrated in Figure 6C, the OPLS-DA score plot revealed distinct clustering and separation trends among sample groups. Model validity was confirmed through 200 permutation tests, where post-permutation *R*^2^ and *Q*^2^ values were significantly lower than those of the original model (*p* < 0.05), demonstrating no overfitting and robust discriminatory power (Figure 6D). Based on the OPLS-DA model, differential metabolites were identified using criteria of variable importance in projection (VIP) > 1, absolute fold change |FC| > 2, and *p* < 0.05 (Figure 7).

For the LC–MS platform, compared to the Control group, the Model group exhibited significant downregulation of D-Xylose, Epicoprostanol, 5-Dodecanol, 2-Deoxy-D-Erythro-Pentitol, Xylitol, Xylose, and Erythronic Acid, while Imazamox, Chenodeoxycholic Acid, 2-Phenylindole, Eicosapentaenoic Acid, Methyl 18-Hydroxyoctadec-9-Enoate, Nonaethylene Glycol, Undecaethylene Glycol, Fructose 1-Phosphate, Tetrahydrocannabinol, 5*α*-Androstan-3α-Ol-17-One, Norethindrone, and 10Z-Nonadecenoic Acid were significantly upregulated. Notably, interventions with 1-DNJ, TFs, and 1-DNJ + TFs modulated Galactaric Acid, Beta-D-Glucose 6-Phosphate, Beta-Glycerophosphoric Acid, Spermidine, and Succinylacetone levels.

For the GC–MS platform, 17 metabolites were significantly upregulated in the Model group relative to the Control group, including 7-Sulfocholic acid, Cichorioside N, Goshonoside F5, DG(PGD2/2:0/0:0), DG(8:0/6 keto-PGF1α/0:0), Butyryl trihexyl citrate, Octaethylene glycol, 6-Ketoprostaglandin E1, 7-Acetoxy-2E,4E,6E-tetradecatrienoic acid, DG(2:0/20:3(8Z,11Z,14Z)-2OH(5,6)/0:0), Mupirocin, PC(LTE4/22:1(13Z)), Phytyl 2-O-methyl-dimethylarsinoylribose, 20-dihydroxyleukotriene B4, (2R,4S)-4-Carbamimidamido-3-acetamido-2-((1R,2R)-2-hydroxy-1-methoxy-3-(octanoyloxy)propyl)-3,4-dihydro-2H-pyran-6-carboxylic acid, 4-(5′-((1E,3E,6Z,9Z)-dodeca-1,3,6,9-tetraen-1-yl)-[3,3′-bi(1,2-dioxolan)]-5-yl)-4-hydroperoxybutanoic acid, and 3-O-α-L-Rhamnopyranosyl-3-hydroxy-5Z-tetradecenoyl-3-hydroxydecanoic acid. Downregulated metabolites included (±)-(Z)-2-(5-Tetradecenyl)cyclobutanone, Tetracosahexaenoic acid, and LysoPE(0:0/15:0). Further analysis revealed: 1-DNJ intervention reduced Goshonoside F5 and DG(PGD2/2:0/0:0) levels; TFs intervention upregulated LysoPE (0:0/15:0) while suppressing Octaethylene glycol; combined 1-DNJ + TFs treatment synergistically regulated LysoPE (0:0/15:0) and Goshonoside F5 levels. These findings suggest that 1-DNJ and TFs may synergistically ameliorate T2DM through coordinated modulation of common metabolic pathways.

**Figure 8:**
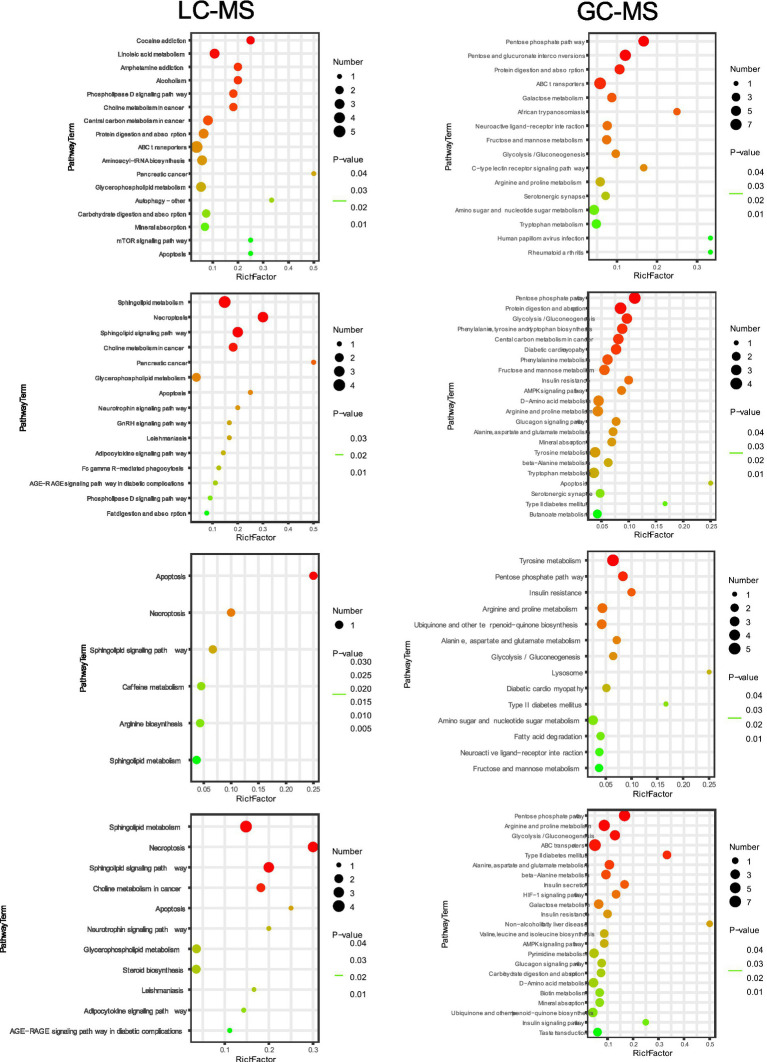
KEGG enrichment pathway map of differentially abundant metabolites.

Pathway enrichment analyses were independently performed for LC–MS and GC–MS datasets to elucidate the biological significance of differential metabolites using the KEGG database. For the LC–MS platform, metabolic pathways were visualized through bubble plots (Figure 8). In the plot, the y-axis represents pathway names (Pathway Term), the x-axis indicates enrichment factors (Rich Factor), bubble color intensity reflects *p*-value significance, and bubble size corresponds to the ratio of differential metabolite counts to species annotation background. Results showed that Model group differential metabolites were significantly enriched in cocaine addiction, linoleic acid metabolism, amphetamine addiction, phospholipase D signaling pathway, central carbon metabolism in cancer, choline metabolism, protein digestion and absorption, ABC transporters, aminoacyl-tRNA biosynthesis, and glycerophospholipid metabolism pathways. Notably, following interventions with 1-DNJ, TFs, and 1-DNJ + TFs, differential metabolites primarily targeted sphingolipid metabolism, necroptosis, sphingosine signaling pathway, and glycerophospholipid metabolism, suggesting these pathways may represent critical regulatory nodes for IR intervention. For the GC–MS platform, Model group differential metabolites were significantly enriched in pentose phosphate pathway, pentose and glucuronate interconversions, protein digestion and absorption, ABC transporters, galactose metabolism, African trypanosomiasis, neuroactive ligand-receptor interaction, fructose and mannose metabolism, glycolysis/gluconeogenesis, arginine and proline metabolism, aminosugar and nucleotide sugar metabolism, and tryptophan metabolism pathways. Intervention experiments further revealed: common regulation by 1-DNJ, TFs, and combined treatment occurred in pentose phosphate pathway, glycolysis/gluconeogenesis, insulin resistance, glucagon signaling pathway, arginine and proline metabolism, fructose and mannose metabolism, and IR pathways; 1-DNJ-specific regulation involved protein digestion and absorption, amino acid biosynthesis/metabolism, central carbon metabolism in cancer, AMPK signaling pathway, mineral absorption, and butanoate metabolism; TFs-specific regulation included ubiquinone and other terpenoid-quinone biosynthesis, lysosome, apoptosis, fatty acid degradation, and neuroactive ligand-receptor interaction pathways; synergistic regulation by 1-DNJ + TFs occurred in ABC transporters, HIF-1*α* signaling pathway, AMPK signaling pathway, carbohydrate digestion and absorption, mineral absorption, and ubiquinone and other terpenoid-quinone biosynthesis pathways. Collectively, LC–MS and GC–MS data suggest that 1-DNJ and TFs may synergistically improve IR metabolic dysregulation through coordinated targeting of sphingolipid metabolism, glycerophospholipid metabolism, glycolysis/insulin resistance pathways, and key signaling cascades such as AMPK signaling.

**Figure 9:**
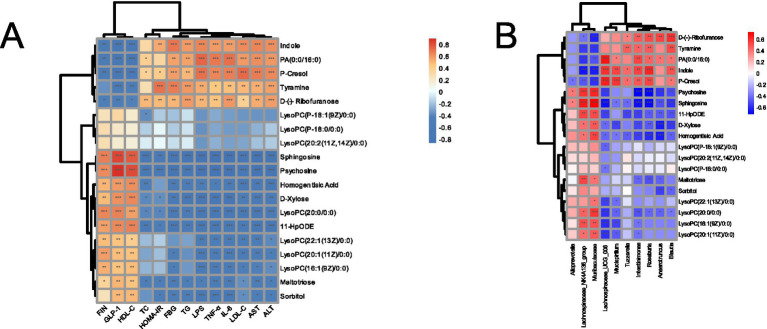
**(A)** Correlation heatmap between differentially abundant metabolites and biochemical indicators. **(B)** Correlation heatmap between differentially abundant metabolites and differentially abundant bacteria.

A Spearman correlation analysis was further performed to evaluate associations between metabolites and serum biochemical indices/intestinal microbiota abundance, aiming to identify potential biomarkers for 1-DNJ combined with TFs in improving T2DM. As shown in Figure 9A, sorbitol, maltotriose, D-xylose, sphingosine, galactosylsphingosine, homogentisic acid, 12,13-DHOME, 11-hydroperoxyoctadecadienoic acid, LysoPC(16:1(9Z)/0:0), LysoPC(20:1(11Z)/0:0), and LysoPC(22:1(13Z)/0:0) showed negative correlations with LDL-C, AST, ALT, LPS, TNF-α, and IL-6, but positive correlations with GLP-1, HDL-C, and insulin levels. Indole, D-(−)-ribofuranose, tyramine, p-cresol, and PA (0:0/16:0) exhibited positive correlations with LDL-C, AST, ALT, LPS, TNF-α, and IL-6, as well as positive associations with HOMA-IR, while demonstrating negative correlations with GLP-1, HDL-C, and insulin levels. LysoPC(P-18:0/0:0), LysoPC(P-18:1(9Z)/0:0), and LysoPC(20:2(11Z,14Z)/0:0) showed no significant correlations with serum biochemical parameters.

The correlations between metabolites and intestinal microbiota are presented in Figure 9B. Levels of LysoPC(20:1(11Z)/0:0), LysoPC(20:0/0:0), LysoPC(16:1(9Z)/0:0), D-xylose, sphingosine, galactosylsphingosine, homogentisic acid, and 11-hydroperoxyoctadecadienoic acid were positively correlated with the abundances of *Muribaculaceae and Lachnospiraceae_NK4A136_group*, but negatively correlated with *Lachnospiraceae_UCG_006* and *Mucispirillum*. Additionally, D-xylose, sphingosine, galactosylsphingosine, homogentisic acid, and 11-hydroperoxyoctadecadienoic acid showed negative associations with *Anaerotruncus, Blautia, Intestinimonas, Roseburia,* and *Tuzzerella*. Indole, D-(−)-ribofuranose, tyramine, p-cresol, and PA (0:0/16:0) were negatively correlated with Muribaculaceae and Lachnospiraceae_NK4A136_group, but positively associated with *Anaerotruncus, Blautia, Intestinimonas, Mucispirillum, Roseburia, Tuzzerella*, and *Lachnospiraceae_UCG_006*. These results indicate that these key metabolites were linked to the therapeutic effects of 1-DNJ combined with TFs in improving T2DM, and intestinal microbiota are associated with these critical metabolic signatures.

## 4 Discussion

Current research has established that both 1-DNJ and TFs possess well-documented hypoglycemic effects. Specifically, 1-DNJ selectively inhibits *α*-glucosidase activity, effectively delaying intestinal glucose absorption and reducing postprandial blood glucose levels (8). TFs exhibit multifaceted glucose-regulatory functions through multiple mechanisms, including digestive enzyme inhibition, insulin pathway activation, and stimulation of intestinal incretin secretion (66–68). IR is the core pathophysiological basis of diseases such as T2DM, obesity, and metabolic syndrome, and its occurrence is closely associated with nutritional excess, especially an HFD. Building upon previous research, this study evaluated the synergistic effects of 1-DNJ and TFs co-intervention using a high-fat diet-induced IR mice model. Results demonstrated that combined 1-DNJ and TFs intervention outperformed monotherapy in multiple aspects: slowing body weight gain, reducing FBG levels, improving glucose tolerance, attenuating systemic inflammation and IR, lowering blood lipid levels, and ameliorating hepatic function parameters and inflammatory markers.

The results of this study demonstrated that the combined use of 1-DNJ and TFs exerted a synergistic effect in reducing the levels of pro-inflammatory cytokines TNF-α and IL-6 in colonic tissues, while also significantly decreasing the LPS level in serum. This finding is consistent with the reports in existing literature regarding the individual anti-inflammatory effects of 1-DNJ and theaflavins. Recent studies have indicated that 1-DNJ can effectively reduce the levels of inflammatory factors such as TNF-α and IL-6 in obese mouse models by inhibiting the IKKβ/NF-κB signaling pathway (19). Similarly, TFs have also been demonstrated to significantly inhibit the secretion of TNF-α and IL-6 by LPS-induced macrophages *in vitro*, and their mechanism of action may be associated with the suppression of TLR4 gene expression as well as the LPS/TLR4 signaling pathway (20). GLP-1, a crucial incretin secreted by intestinal L-cells, regulates glucose metabolic homeostasis through mechanisms including insulin secretion promotion, glucagon release suppression, and enhancement of peripheral tissue glucose uptake (21). This study revealed that combined 1-DNJ and TFs intervention synergistically restored GLP-1 levels to normal ranges in IR mice, suggesting protective effects on enteroendocrine L-cell function. The level of LPS in serum was decreased, along with the up-regulated expression of intestinal tight junction proteins ZO-1, Claudin-1, and Mucin 2. This is consistent with the reports in existing literature that high-fat diet impairs the intestinal barrier (22, 23).

Observed correlations between reduced serum LPS and diminished inflammatory markers suggest pivotal involvement of gut-derived inflammation regulation in metabolic improvements (24). Recent studies demonstrated that gut microbiota dysbiosis exacerbates T2DM progression through multiple mechanisms. Abnormal elevation of the Firmicutes/Bacteroidetes ratio increases LPS production, while microbial metabolites disrupt tight junction proteins, triggering gut-derived inflammatory cytokine storms (25–28). Microbiota transplantation experiments confirm the transmissible nature of T2DM phenotypes via gut flora, whereas healthy donor microbiota transplantation significantly improves glucose-lipid metabolism (29).

Epidemiological studies have revealed that the correlations between microbial compositional changes and key pathological mechanisms including insulin deficiency, insulin resistance, and low-grade inflammation (30–34). Gut microbiota analysis demonstrated decreased microbial richness in the Model group compared to Control, accompanied by structural microbial community shifts. Although no statistically significant differences in microbial richness and diversity were observed among the 1-DNJ, TFs, and 1-DNJ + TFs groups compared to Model group, a trend toward richness restoration was noted. The fecal microbial profiles of all intervention groups (1-DNJ, TFs, and 1-DNJ + TFs) showed significant structural divergence from Model group while approaching Control group patterns.

Consistent with published reports (35), Model group exhibited characteristic phylum-level dysbiosis versus Control: increased *Firmicutes* and *Desulfobacterota* abundances accompanied by reduced *Bacteroidota* proportions, along with decreased microbial richness. Following 1-DNJ, TFs, and combined interventions, we observed progressive *Bacteroidota* restoration and Firmicutes reduction, with the 1-DNJ + TFs combination further increasing *Proteobacteria* abundance. At genus level, Model group displayed decreased *Muribaculaceae* and Lachnospiraceae_NK4A136_group abundances alongside elevated *Mucispirillum, Roseburia, Colidextribacter, Helicobacter, Lachnoclostridium*, and *Blautia* populations. All three intervention regimens demonstrated trends toward normalization of these bacterial taxa. LEfSe analysis of dominant microbiota across groups revealed that the predominant bacterial taxa in T2DM mice included *Helicabacter, Blautia, Roseburia, Colidextribacter, Intestinimonas*, and *Anaerotruncus*, whereas *Alloprevotella* and *Bacteroides* were significantly reduced in T2DM mice. Previous studies have reported increased abundance of *Blautia* in obese T2DM mice, consistent with the findings of this study (36). An epidemiological investigation further corroborated that elevated *Blautia* abundance is associated with T2DM pathogenesis (37). *Helicabacter, Colidextribacter*, and *Anaerotruncus* are recognized pathogenic genera linked to inflammation, lipid metabolism dysregulation, and oxidative stress (38).

Notably, *Colidextribacter* has been shown to exhibit a positive correlation with fasting blood glucose levels. Following 1-DNJ intervention, alterations were observed in D*ubosiella, Erysipelotrichaceae, Lachnospiraceae_UCG_010, Tuzzerella, UCG_009*, and *Oscillibacter*. Similarly, TFs intervention induced changes in *Erysipelatoclostridium*, *Enterorhabdus, Faecalibaculum, Eubacterium__coprostanoligenes_group, Clostridia, Eubacterium_nodatum_group, Oscillospirales,* and *Coriobacteriale*s. The combined 1-DNJ + TFs intervention significantly modulated T*annerellaceae, Anaerovoracaceae, Lachnoclostridium, Bacteroides, Alloprevotella, Rikenella, Parabacteroides*, and *Enterobacter*. Increasing their abundance plays a pivotal role in ameliorating T2DM pathogenesis, as evidenced by prior studies (39, 40). Compared to healthy individuals, T2DM patients exhibited markedly reduced abundances of *Alloprevotella* and *Bacteroides*—both recognized as key SCFA-producing taxa. Increasing the abundance of these taxa plays a pivotal role in ameliorating T2DM pathogenesis, as evidenced by prior studies (39, 40). Changes in the gut microbiota composition were characterized by an increase in beneficial bacteria (such as *Akkermansia, Bifidobacterium, and Lactobacillus*) and a decrease in certain potentially harmful microbial groups (such as *Enterococcaceae and Lachnospiraceae*) (11, 12). These compositional shifts coincide with observed improvements in glucose and lipid metabolism parameters.

SCFAs metabolic products generated by gut microbiota through the fermentation of dietary fiber and resistant starch, enhance hepatic glycogen synthesis and may regulate blood glucose levels via GLP-1-mediated insulin secretion (41). *Muribaculum* and *Parabacteroides* were also recognized as SCFA-producing taxa (42). In this study, the combined 1-DNJ and TFs intervention significantly increased the abundance of *Muribaculum* and *Parabacteroides*. Furthermore, endogenous metabolites such as indoleacrylic acid produced by Parabacteroides activate the aryl hydrocarbon receptor (AhR) signaling pathway and elevate interleukin-22 (IL-22) expression, thereby enhancing the production of intestinal barrier-associated proteins (43).

Impaired glucose metabolism regulation is associated with the onset and progression of T2DM. Gluconeogenesis, the glucose/glucose-6-phosphate cycle, glycogenolysis/glycogenesis, and the pentose phosphate pathway represent critical processes in maintaining systemic glucose homeostasis (44). Compared to the Control group, metabolites enriched in pathways including the pentose phosphate pathway, gluconeogenesis, and glycogenolysis/glycogenesis - specifically gluconic acid, *β*-D-glucose 6-phosphate, D-(−)-ribofuranose, D-glucose, β-D-fructose 6-phosphate, and pyruvate - exhibited significant alterations in Model group mice. Following 1-DNJ and TFs interventions, D-(−)-ribofuranose, β-D-fructose 6-phosphate, and pyruvate demonstrated trends toward normalization. The 1-DNJ + TFs combination not only significantly elevated these three metabolites but also modulated levels of D-glucose, glucose, and D-ribose. Furthermore, pyruvate showed associations with insulin resistance, glucagon signaling pathways, and IR-related pathways. These findings indicate that 1-DNJ combined with TFs enhances glucose metabolism to ameliorate IR. For decades, the role of lipids in T2DM-associated insulin resistance and pancreatic *β*-cell dysfunction has attracted considerable attention. Excessive free fatty acids, for instance, can lead to dysfunction in insulin-sensitive tissues and pancreatic *β*-cells, disrupt systemic glucose homeostasis, and ultimately trigger T2DM (44). Lipids encompass fatty acids, phospholipids, and sterols. 11-Hydroperoxyoctadecadienoic acid, a derivative of linoleic acid, has been epidemiologically demonstrated to exhibit an inverse correlation with diabetes risk (45). Canetti et al. (46) revealed that linoleic acid protects pancreatic tissue, thereby enhancing insulin production. The 1-DNJ + TFs combination significantly upregulated 11-hydroperoxyoctadecadienoic acid levels, indicating that linoleic acid metabolism represents a crucial pathway for its anti-T2DM effects. Prostaglandin E2 (PGE2), a cyclooxygenase metabolite of arachidonic acid, may contribute to β-cell dysfunction and destruction, potentially participating in the pathogenesis of diabetes and its complications (47). Notably, 1-DNJ + TFs downregulated PGE2 levels. Phospholipids, defined as phosphate-containing lipids, were categorized into glycerophospholipids and sphingomyelins: glycerol-based phospholipids are termed glycerophospholipids, while sphingosine-based ones were sphingomyelins (48). Epidemiological studies have linked glycerophospholipid dysregulation to T2DM pathogenesis (49). In this study, compared with the Control group, the Model group exhibited altered levels of glycerophospholipids including lysophosphatidylcholine (20:1(11Z)/0:0), lysophosphatidylcholine (16:1(9Z)/0:0), and PA (0:0/16:0), indicating disrupted glycerophospholipid metabolism. The 1-DNJ + TFs combination effectively restored these glycerophospholipid levels. Emerging evidence connects sphingolipid metabolism with neurodegenerative diseases (50), obesity (51), and endocrine disorders (52) including diabetes. Epidemiological studies report elevated ceramide and sphingomyelin levels in T2DM patients, showing positive correlations with insulin resistance and BMI (53, 54), suggesting sphingolipid involvement in T2DM pathophysiology. Sphingolipids influence pancreatic β-cell viability; ceramide, a key mediator in T2DM, suppresses insulin signaling and induces β-cell apoptosis (55), whereas sphingosine exerts opposing effects on insulin-sensitive tissues and β-cells (56). Ceramide, sphingomyelin, and sphingosine—core components of sphingolipid metabolism—participate in cellular processes including signal transduction, proliferation, apoptosis, and differentiation (57). In this study, the Model group showed increased sphingomyelin (d18:0/16:1(9Z)) and ceramide (d18:1/16:0) levels alongside decreased sphingosine compared to Controls. The 1-DNJ + TFs intervention upregulated sphingosine while downregulating sphingomyelin (d18:0/16:1(9Z)) and ceramide (d18:1/16:0). These findings demonstrated that sphingolipid metabolism represents another critical pathway through which 1-DNJ + TFs ameliorates T2DM.

Abnormal forms of cell death are associated with various diseases and tissue injuries (58). Necroptosis, a programmed cell death mechanism independent of caspase activation, is mediated through molecules such as receptor-interacting protein kinase 1 (RIPK1), protein kinase 3 (RIPK3), and mixed lineage kinase domain-like protein (MLKL) (59). This process typically occurs when apoptosis is inhibited, and sphingolipid metabolism can indirectly influence necroptosis by modulating apoptotic pathways (60). Furthermore, necroptosis involves alterations in cellular energy metabolism and redox status. NADPH, a critical intracellular antioxidant generated by the pentose phosphate pathway, supplies metabolic intermediates that feed into glycolysis and the tricarboxylic acid (TCA) cycle, thereby impacting cellular energy homeostasis. In arginine and proline metabolism, nitric oxide derived from arginine metabolism can modify proteins via S-nitrosylation, potentially regulating the activity of key necroptosis-related proteins such as RIPK1, RIPK3, and MLKL, thereby influencing necroptotic signaling. Proline, on the other hand, exhibits antioxidant properties. These observations suggested that sphingolipid metabolism, the pentose phosphate pathway, and arginine/proline metabolism may directly or indirectly regulate necroptosis in intestinal enteroendocrine L-cells, consequently affecting GLP-1 secretion.

Emerging evidence highlights that gut microbiota-host cometabolites serve not only as disease biomarkers but also as active participants in disease progression through metabolic regulation during host–microbe interactions. In T2DM and insulin resistance, specific cometabolites including SCFAs, succinate, p-cresol, dimethylglycine, imidazole propionate, tryptophan and its metabolites (kynurenine, indolelactic acid), 1-linoleoyl-glycerophosphocholine, and indole-3-propionic acid exhibit either pathogenic or protective effects. The gut microbiome and its derived metabolites have become a focal point in understanding T2DM pathogenesis. Studies reveal associations between certain gut microbiota and host glucose metabolism, glycerophospholipid metabolism, and sphingolipid metabolism. For instance, Escherichia, Bilophila, Enterorhabdus, Fusobacterium, and Proteus show positive correlations with total glycerophospholipid levels (61, 62), while *Prevotella* (63), *Bacteroides* (64), *Bifidobacterium* (64), and *Bacteroides thetaiotaomicron* (which secretes sphingolipid derivatives) (65) were linked to sphingolipid metabolism. Correlation analyses between metabolites and gut microbiota identify *Muribaculaceae* and *Lachnospiraceae_NK4A136_group* as key microbial taxa associated with elevated levels of LysoPC(20:1(11Z)/0:0), D-xylose, sphingosine, psychosine, homogentisic acid, and 11-hydroperoxyoctadecadienoic acid. Conversely, *Anaerotruncus, Blautia, Intestinimonas, Mucispirillum, Roseburia, Tuzzerella, and Lachnospiraceae_UCG_006* were associated with increased levels of indole, D-(−)-ribofuranose, tyramine, p-cresol, and PA (0:0/16:0). These metabolites primarily participate in sphingolipid metabolism, necroptosis, glycerophospholipid metabolism, the pentose phosphate pathway, pentose and glucuronate interconversions, tyrosine metabolism, and protein digestion/absorption pathways. Notably, protective metabolites (including sorbitol, maltotriose, D-xylose, sphingosine and its derivatives, homogentisic acid, 12,13-DHOME series, and various LysoPCs) demonstrate significant metabolic benefits, showing negative correlations with LDL-C, liver injury markers (AST, ALT), and pro-inflammatory factors (LPS, TNF-α, IL-6), while positively associating with metabolic health indicators like GLP-1 and HDL-C. In contrast, metabolism-disrupting metabolites (e.g., indole, D-ribofuranose, tyramine, p-cresol, and PA) exhibit opposing patterns: positive correlations with HOMA-IR, LDL-C, AST, ALT, LPS, and TNF-α, but negative correlations with protective markers such as GLP-1, HDL-C, and insulin levels. These findings suggested that gut microbiota may exert bidirectional regulatory effects in IR pathogenesis by producing functionally distinct metabolites. Protective metabolites likely confer benefits via anti-inflammatory actions and insulin sensitivity improvement, whereas metabolism-disrupting metabolites may exacerbate inflammation and promote insulin resistance. This metabolic perspective provides novel insights into the molecular mechanisms underlying gut microbiota-mediated influences on T2DM development.

Meanwhile, this study has some limitations: First, the demonstration of intestinal barrier injury mainly relies on indirect evidence at the molecular biology level, while direct evidence for the functional integrity assessment at the tissue or *in vivo* level is lacking. Secondly, although we revealed significant changes in gut microbiota composition and metabolic profiles through 16S rRNA gene sequencing and metabolomic analysis of intestinal contents, we failed to directly quantify the levels of SCFAs, such as acetic acid and butyric acid. As important metabolites of gut microbiota, SCFAs play a crucial role in regulating host energy metabolism, inflammatory responses, and intestinal barrier function. Therefore, the lack of direct measurement of SCFAs limits our in-depth clarification of the mechanism by which the intervention improves IR through influencing these key metabolites. Third, this study identified significantly differential gut microbiota and enriched functional pathways (e.g., results of KEGG analysis) via bioinformatics methods, which provide valuable clues for mechanism exploration. However, we have not yet verified the causal relationships and specific contributions of these differential microbiota and target pathways to the improvement of IR through functional experiments, such as animal fecal microbiota transplantation (FMT) or targeted intervention. Future studies may consider conducting FMT experiments, in which gut microbiota from treated mice are transplanted into germ-free or model mice, to verify the ameliorative effect of specific microbiota on host metabolism. Meanwhile, performing *in vitro* or in vivo functional verification of the screened differential strains and key pathways will help to more precisely elucidate their roles in the efficacy of combined intervention.

## 5 Conclusion

In conclusion, this study revealed that the combined intervention of 1-DNJ and TFs exhibited superior efficacy in ameliorating IR compared to monotherapy with individual components. The combined intervention modulated the gut microbiota by enhancing the abundance of beneficial bacteria, including *Muribaculaceae*, *Lachnospiraceae_NK4A136_group*, and *Alloprevotella*, while simultaneously reducing harmful bacterial genera such as *Roseburia* and *Intestinimonas*. Concurrently, these microbial shifts were associated with significant modulations in various intestinal metabolic pathways, encompassing sphingolipid metabolism, necroptosis, glycerophospholipid metabolism, the pentose phosphate pathway, pentose and glucuronic acid interconversion, and tyrosine metabolism.

## Data Availability

The datasets presented in this study can be found in online repositories. The names of the repository/repositories and accession number(s) can be found in the article/supplementary material.

## Glossary

T2DM: Type 2 Diabetes Mellitus
HFD: High-Fat Diet
LPS: Lipopolysaccharide
IL-2: Interleukin-2
ZO-1: Zonula Occludens-1
TNF-α: Tumor Necrosis Factor-alpha
IL-6: Interleukin-6
AST: Aspartate Aminotransferase
ALT: Alanine Aminotransferase
LDL-C: Low-Density Lipoprotein Cholesterol
HOMA-IR: Homeostatic Model Assessment for Insulin Resistance
HDL-C: High-Density Lipoprotein Cholesterol
GLP-1: Glucagon-Like Peptide-1
IR: Insulin Resistance
FBG: Fasting Blood Glucose
IPGTT: Intraperitoneal Glucose Tolerance Test
AUC: Area Under the Curve
TCTG: Total Cholesterol and Triglycerides
LC–MS: Liquid Chromatography-Mass Spectrometry
GC–MS: Gas Chromatography–Mass Spectrometry
16 s rRNA: 16S ribosomal RNA
